# The role of resection in hepatocellular carcinoma BCLC stage B: A multi-institutional patient-level meta-analysis and systematic review

**DOI:** 10.1007/s00423-024-03466-x

**Published:** 2024-09-13

**Authors:** Victor Lopez-Lopez, Fabian Kalt, Jian-Hong Zhong, Cristiano Guidetti, Paolo Magistri, Fabrizio Di Benedetto, Arndt Weinmann, Jens Mittler, Hauke Lang, Rohini Sharma, Mathew Vithayathil, Samir Tariq, Patricia Sánchez-Velázquez, Gianluca Rompianesi, Roberto Ivan Troisi, Concepción Gómez-Gavara, Mar Dalmau, Francisco Jose Sanchez-Romero, Camilo Llamoza, Christoph Tschuor, Uluk Deniz, Georg Lurje, Peri Husen, Sandro Hügli, Jan Philipp Jonas, Fabian Rössler, Philipp Kron, Michaela Ramser, Pablo Ramirez, Kuno Lehmann, Ricardo Robles-Campos, Dilmurodjon Eshmuminov

**Affiliations:** 1 Department of General, Visceral and Transplantation Surgery, Clinic and University Hospital Virgen de La Arrixaca, IMIB-ARRIXACA, El Palmar, Murcia, Spain; 2 Department of Surgery and Transplantation, University Hospital Zurich and University of Zurich, Raemistrasse 100, Zurich, CH-8091 Switzerland; 3 Department of Hepatobiliary Surgery, Guangxi Medical University Cancer Hospital, Nanning, 530021 China; 4 Hepato-Pancreato-Biliary Surgery and Liver Transplantation Unit, University of Modena and Reggio Emilia, Modena, Italy; 5 Department of Internal Medicine I, University Medical Center of the Johannes Gutenberg University Mainz, Mainz, Germany; 6 Department of General, Visceral and Transplant Surgery, University Medical Center, Johannes Gutenberg-University Mainz, 55131 Mainz, Germany; 7 Department of Surgery and Cancer, Imperial College London, London, UK; 8 Division of Hepatobliary and pancreatic Surgery, Hospital del Mar, Universitat Pompeu Fabra, IMIM, Barcelona, Spain; 9 Department of Clinical Medicine and Surgery, Federico II University, Naples, Italy; 10 Department HPB and Transplantation Surgery, Hospital Vall d’Hebron, Universitat Autònoma de Barcelona, Barcelona, Spain; 11 Department of Surgery and Transplantation, Rigshospitalet Copenhagen University Hospital, Blegdamsvej 9 Copenhagen Ø, Copenhagen, 2100 Denmark; 12 Department of Surgery, Charité – Universitätsmedizin Berlin, corporate member of Freie Universität Berlin, Humboldt-Universität zu Berlin, Berlin Institute of Health, Berlin, Germany; 13 Department of General, Visceral and Transplantation Surgery, Heidelberg University Hospital, Heidelberg, Germany

**Keywords:** HCC, BCLC-B, Liver resection, Liver transplantation

## Abstract

**Purpose:**

The Barcelona Clinic Liver Cancer (BCLC) staging schema is widely used for hepatocellular carcinoma (HCC) treatment. In the updated recommendations, HCC BCLC stage B can become candidates for transplantation. In contrast, hepatectomy is currently not recommended.

**Methods:**

This systematic review includes a multi-institutional meta-analysis of patient-level data. Survival, postoperative mortality, morbidity and patient selection criteria for liver resection and transplantation in BCLC stage B are explored. All clinical studies reporting HCC patients with BCLC stage B undergoing liver resection or transplantation were included.

**Results:**

A total of 31 studies with 3163 patients were included. Patient level data was available for 580 patients from 9 studies (423 after resection and 157 after transplantation). The overall survival following resection was 50 months and recurrence-free survival was 15 months. Overall survival after transplantation was not reached and recurrence-free survival was 45 months. The major complication rate after resection was 0.11 (95%-CI, 0.0-0.17) with the 90-day mortality rate of 0.03 (95%-CI, 0.03–0.08). Child-Pugh A (93%), minor resection (60%), alpha protein level less than 400 (64%) were common in resected patients. Resected patients were mostly outside the Milan criteria (99%) with mean tumour number of 2.9. Studies reporting liver transplantation in BCLC stage B were scarce.

**Conclusion:**

Liver resection can be performed safely in selected patients with HCC BCLC stage B, particularly if patients present with preserved liver function. No conclusion can done on liver transplantation due to scarcity of reported studies.

**Supplementary Information:**

The online version contains supplementary material available at 10.1007/s00423-024-03466-x.

## Introduction

Hepatocellular carcinoma (HCC), the most common primary liver cancer, is staged using various systems [1]. Among these, the Barcelona Clinic Liver Cancer (BCLC) schema, continuously refined, recently underwent its latest update in 2022, and remains instrumental in guiding HCC treatment decisions due to its incorporation of tumor burden, liver function, and performance status [2–5]. 

The updated BCLC recommendation allows liver transplantation for selected BCLC stage B patients, reflecting current evidence that it offers the best chance for long-term cure, especially considering potential underlying liver disease [3]. However, organ scarcity limits its availability [6]. Notably, these updated guidelines discourage using hepatectomy in BCLC stage B, leaving locoregional or palliative options as alternatives [3]. 

Despite the widespread acceptance of the BCLC system, its treatment algorithm for BCLC stage B has been challenged by some groups due to the poor prognosis of non-curatively treated patients (median survival < 18 months) and the potential benefits of liver resection in this stage, as evidenced by previous publications [2, 7–9]. 

However, the suitability of liver resection for BCLC stage B remains a matter of debate. This systematic review and meta-analysis aims to address this critical gap by summarizing existing evidence on outcomes after liver resection or transplantation in these patients. We analyzed survival, perioperative mortality and morbidity, and patient selection criteria for BCLC stage B resections. For survival analysis patient level data was used, provided by authors upon personal communication.

## Methods

### Study search and selection

This meta-analysis was conducted following the Preferred Reporting Items for Systematic Reviews and Meta-analysis (PRISMA) statement [10]. The protocol was registered prior to search in the PROSPERO database (CRD42020172986). The systematic search was conducted on the databases of Medline, Scopus, Embase and Cochrane for clinical studies in English without time restrictions. Additional studies were searched also manually including the reference list of included studies. All single arm clinical studies reporting HCC patients with BCLC stage B undergoing liver resection or transplantation were included. Animal studies, conference abstracts, case series with less than 10 patients, letters, reviews, study protocols and other type of non-original articles were excluded. The initial search was conducted on July 2, 2020 (details in Supplementary file 1), with a manual update performed on June 17, 2024.

### Data collection and measures

Two independent reviewers (V.L.L. and F.K.) screened the identified abstracts based on relevance. Data extraction was performed using Microsoft Excel. Any discrepancies identified during the review process were resolved through discussion among the reviewers. For complex issues, the senior author was consulted to ensure a consensus. Studies definitively meeting the predefined inclusion and exclusion criteria were included in the meta-analysis after full-text assessment. The following data were extracted for analysis: study design, study population, BCLC stage, treatment (liver resection or transplantation), MELD score, Child-Pugh class, Milan criteria, lesion number and size, AFP level, morbidity, and 90-day mortality. Survival analysis was conducted using patient-level data from eight centers. Other outcome analyses utilized data extracted from included studies and any available patient-level data, when applicable. Overall survival was the primary endpoint, with secondary outcomes encompassing recurrence-free survival, perioperative morbidity, perioperative mortality, and selection criteria for liver transplantation or resection. Qualitative synthesis was utilized for non-quantitative findings to draw conclusions. To assess the quality of retrieved evidences a GRADE assessment was used [11]. Risk of bias was assessed by Newcastle–Ottawa quality assessment scale [12]. 

### Statistics

The meta-analysis of dichotomous data in single group data was conducted using the rBiostatistics with random-effects meta-analysis due to expected heterogeneity of the data. The results were reported in rates with 95% confidence interval (CI). The heterogeneity was evaluated with I2 statistics according to Cochrane Handbook [13]. I2 statistics was interpreted as following: 0-40% not important; 30-60%: moderate heterogeneity; 50-90% substantial heterogeneity; 75-100% considerable heterogeneity. *P* values was used to evaluate I2 results. No transformation was implemented in proportion of 0 in analysis. SPSS (version 25) was used for analysis of survival outcomes with the Kaplan-Meier method.

## Results

### Included studies and descriptive data

**Fig. 1:**
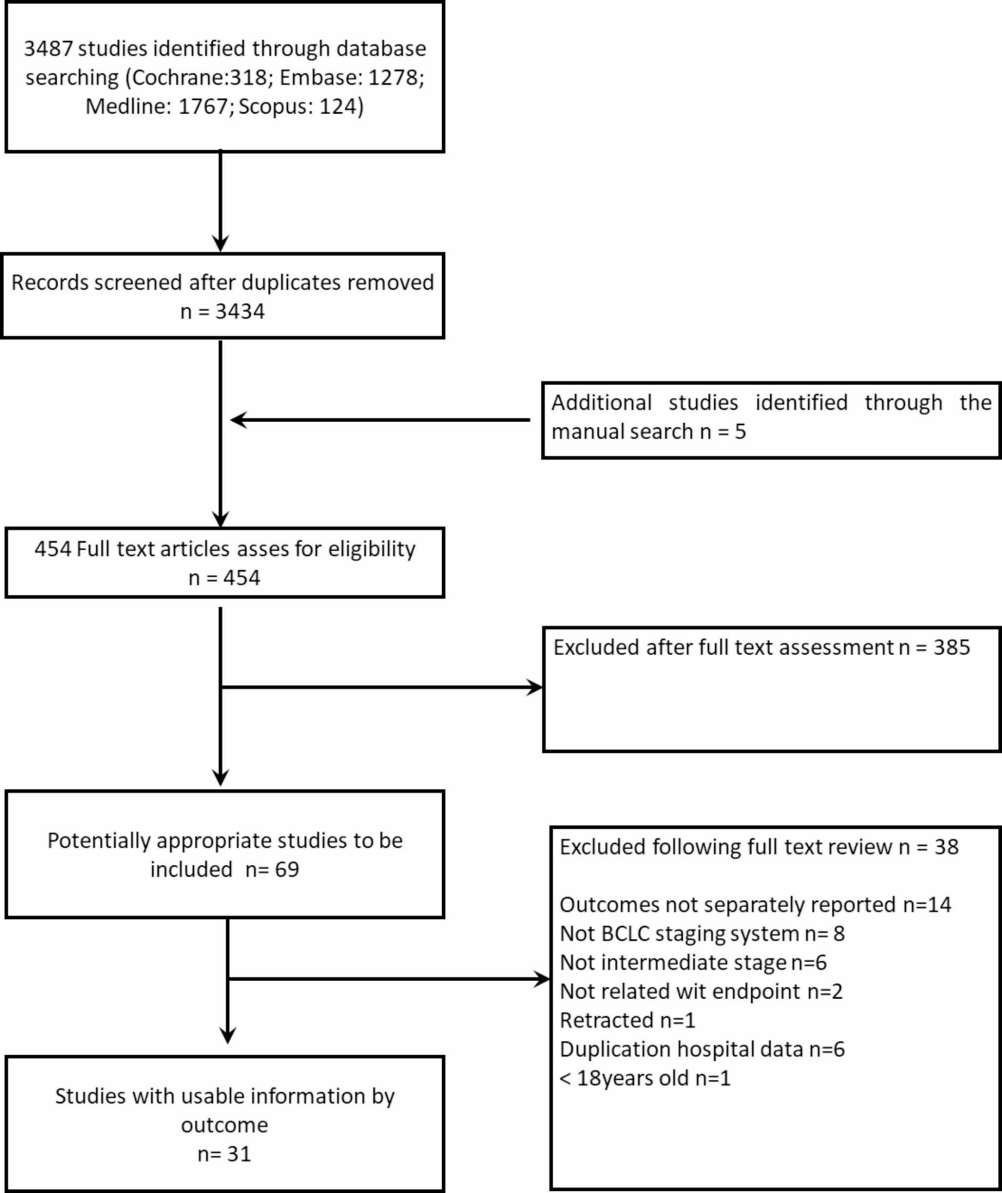
Flowchart of study selection

Our systematic search identified 3434 studies after removing duplicates. The detailed selection process along with the specific reasons for inclusion or exclusion are presented in Fig. 1. Ultimately, 31 studies encompassing 3163 patients were included in the final analysis [12–40]. Of these, 9 studies provided patient-level data for 580 patients (423 after resection and 157 after transplantation) obtained directly from authors through personal communication. The remaining 22 studies lacked patient-level data, with 21 reporting outcomes solely on liver resection and one solely on liver transplantation. It is important to note that all included studies were retrospective (Table 1). The evidence level accordingly to the GRADE approach is available online as Supplementary Table 2. The publication bias according to Newcastle–Ottawa quality assessment scale was reported in Supplementary Table 3.

**Table 1:** Included studies. Tumor number and size were reported in mean with standard deviation or in median with range. The patient level data were available for the last 9 included studies (from Berardi et al.). The data from Charité were provided upon personal communication.

Author / Year	Patients	HCC Child A	Tumor size	Tumor number	AFP level
Bell et al. 2016 [17]	17 / resection	17			
Di Sandro et al. 2019 [18]	131 / resection	110			17 (5-316)
Fang et al. 2019 [19]	104 / resection				
Garancini et al. 2017 [20]	24 / resection	22	5.3 (1.1–16)	2 (1–5)	
Lei et al. 2014 [21]	433 / resection	328	7 (6–8)	2 (1–3)	
Kamiyama et al. 2017 [22]	297 / resection	290			
Kamo et al. 2018 [23]	12 / transplant				6 (2-186)
Kariyama et al. 2020 [24]	165 / resection	155			
Kim H et al. 2017 [25]	83 / resection	79	5 (1.9–13.6)	2 (2–7)	
Kim J et al. 2016 [26]	52 / resection	51			
Lin C. T. et al. 2010 [27]	93 / resection	93	8 (3.3)		
Lin C. W. et al. 2020 [28]	140 / resection	134	8.2 (3.3)		
Liu Y. et al. 2020 [29]	73 / resection	72	5.8(4.2–8.4		
Matsukuma et al. 2018 [30]	65 / resection	64	4 (1.5–17)	3	32 (0.8-239621)
Peng et al. 2019 [31]	70 / resection	67	5.0(3.0-15.5)		
Renner et al. 2015 [32]	46 / resection				7.8 (3-119)
Torzilli et al. 2008 [33]	24 / resection		5 (2–28)	2 (1–6)	13 (2–62)
Tsilimigras et al. 2019 [34]	180 / resection	177			
Wada et al. 2016 [35]	85 / resection	75	5.7 (2.2)		39 (3-312)
Wang et al. 2016 [36]	78 / resection	76	5 (2.5–20)	17.8 (1.1–1211.0)	
Wei S. et al. 2011 [37]	51 / resection				
Wei W. et al. 2018 [38]	360 / resection	332	5.2 (4–8)		
Berardi et al. 2019 [39]	16 / resection	15	6.4 (5)	2.6 (1.2)	5.2 (3-450)
Di Benedetto et al. 2023 [40]	97 / combined	69	4.3 (2.4)	3.5 (1.4)	11 (6.2–22.8)
Lim et al. 2018 [41]	12 / transplant	7	4.0 (1.5)	4.5 (2.6)	4.9 (3.5–16.2)
Lopez-Lopez et al. 2021 [42]	42 / combined	32	5.2 (2.9)	3.4 (1.4)	7.3 (4.6–14)
Ramasvami et al. 2016 [43]	23 / resected	18	5.1 (3)	3.7 (1.4)	27.5 (5-315)
Villamonte et al 2022 [44]	15 / resected	12	3 (0)	2.4 (0.5)	8.3 (1.7–410)
Weinmann et al. / 2015 [45]	59 / combined	45	6.9 (3.8)	3.4 (1.4)	8.2 (6–15)
Zhong et al. / 2014 [9]	166 / resected	160	7.4 (3.6)	2.1 (0.7)	248 (147–480)
Charité, Berlin	150 / combined				

### Patient survival after liver resection

**Fig. 2:**
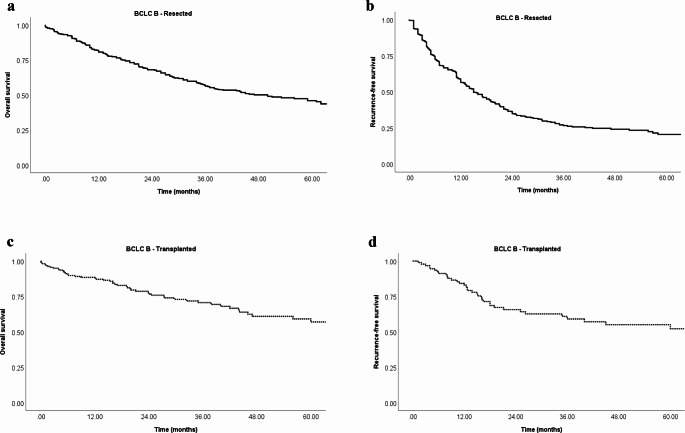
(**a**) Overall survival resected patients, (**b**) Recurrence free survival resected patients, (**c**) Overall survival transplanted patients, (**d**) Recurrence free survival transplanted patients

The median overall survival (OS) after resection was 50 months (95% CI 38–62 months) with a 5-year survival rate of 46% (Fig. 2a) in the patient-level analysis encompassing 423 patients. The patient-level data analysis showed a recurrence-free survival (RFS) of 15 months (95% CI 12–18 months) with a 5-year recurrence-free rate of 21% (Fig. 2b). For studies lacking patient-level data, the reported 5-year survival rate ranged between 30% and 63% after liver resection. Among the 9 studies with patient-level data, the microvascular and macrovascular invasion rates were 0.42 (95% CI, 0.34–0.51; I^2^ = 66%, *p* < 0.01) and 0.08 (95% CI, 0.03–0.21; I^2^ = 63%, *p* = 0.01), respectively. Notably, R0 resection rate was high (0.92; 95%-CI, 0.82–0.97; I^2^ = 83%, *p* < 0.01).

### Morbidity and 90-day mortality after liver resection

**Fig. 3:**
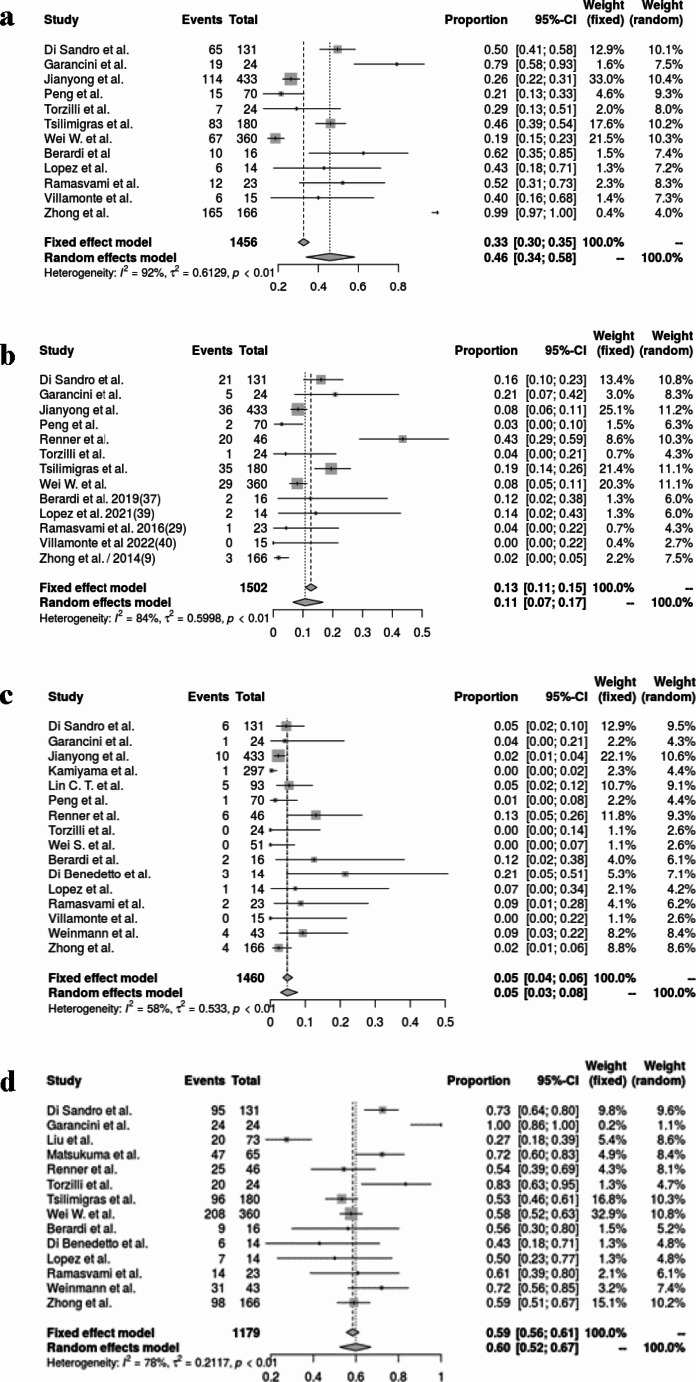
(**a**) Overall complication rate, (**b**) Major complication rate, (**c**) 90 day mortality, (**d**) Minor resection rate

The pooled rate of any complication following liver resection was 0.46 (95% CI, 0.34–0.58; I^2^ = 92%, *p* < 0.01) (Fig. 3a). Similarly, the pooled rate of major complications (Clavien-Dindo grade ≥ 3a) was 0.11 (95% CI, 0.0-0.17; I^2^ = 84%, *p* < 0.01) (Fig. 3b). The analysis from 16 studies revealed a pooled 90-day mortality rate of 0.03 (95% CI, 0.03–0.08; I^2^ = 58%, *p* < 0.01) (Fig. 3c). The most frequent type of resection was minor resection, occurring at a pooled rate of 0.60 (95% CI, 0.5–0.67; I^2^ = 78%, *p* < 0.01) (Fig. 3d).

### Selection for resection in BCLC stage B

Considering that liver resection is uncommon in BCLC stage B, and careful patient selection is crucial, we aimed to analyze the selection criteria used for liver resection in this patient population. Most patients undergoing liver resection had a Child-Pugh score A with rate of 0.93 (95% CI, 0.90–0.96; I^2^ = 90%, *p* < 0.01). Child-Pugh B and a MELD score exceeding 10 were both rare in resected patients (Child-Pugh B: 0.07; 95% CI, 0.04–0.11; ; I^2^ = 90%, *p* < 0.01, MELD score: 7.5; 95% CI, 5.31–9.69; I^2^ = 0, *p* = 0.61). Data from eight studies reported median AFP levels, which did not exceed 70 except in one study. Additionally, 11 studies proposed an AFP cut-off level of 400. In these studies, the rate of patients with AFP levels below 400 was 0.64 (95% CI, 0.58–0.70; I^2^ = 83%, *p* < 0.01). The mean tumor number was 2.88 (95% CI, 1.69–4.07; I^2^ = 0, *p* = 0.85) with a largest tumor size of 6.85 cm (95% CI, 2.96–10.74; I^2^ = 0, *p* = 0.99). Notably, patients who underwent liver resection fell outside the Milan criteria (0.99; 95% CI, 0.95-1; I^2^ = 0, *p* = 0.6).

### Outcome after transplantation

The study also aimed to analyse outcomes after liver transplantation, Unfortunately the authors could identify only one study without patient level data (*n* = 12). The rest was a patient level data for 153 patients obtained after personal communication.

The median overall survival (OS) after transplantation was not reached, with a 5-year survival rate of 57% (Fig. 2c) in 153 patients. Similarly, the median recurrence-free survival (RFS) was not reached, with a 5-year RFS rate of 52% (Fig. 2d). The microvascular and macrovascular invasion rates were 0.11 (95% CI, 0.02–0.44; I^2^ = 69%, *p* = 0.04) and 0.05 (95% CI, 0.01–0.2; I^2^ = 60%, *p* = 0.08), respectively. As expected, all patients achieved an R0 resection margin.

Morbidity and mortality data after liver transplantation was available only from studies with patient-level data. The overall complication rate was 0.57 (95% CI, 0.45–0.68; I^2^ = 0, *p* = 0.41), and the clinically significant major complication rate was 0.13 (95% CI, 0.04–0.36; I^2^ = 63%, *p* = 0.07). The 90-day mortality rate was 0.06 (95% CI, 0.03–0.12; I^2^ = 0, *p* = 0.84).

Transplanted patients revealed a mixed picture regarding the Child-Pugh group, with Child-Pugh A at rate of 0.60 (95% CI, 0.43–0.74; I^2^ = 62%, *p* = 0.05). They had a mean pre-transplant MELD score of 10.4 (95% CI, 5.14–15.63; I^2^ = 0, *p* = 0.85). Notably, the majority of patients received TACE prior to transplantation (0.9; 95% CI, 0.71–0.97; I^2^ = 24%, *p* = 0.4). All transplanted patients were outside the Milan criteria (0.98; 95% CI, 0.93–0.99; I^2^ = 0, *p* = 0.89), with a mean tumor number of 3.19 (95% CI, 1.41–4.96; I^2^ = 0, *p* = 0.84) and a mean largest tumor size of 4.72 cm (95% CI, 1.62–7.82; I^2^ = 0, *p* = 0.94). The mean AFP level in transplanted patients was 56.7 (95% CI, -136.29-249.74; I^2^ = 0, *p* = 0.98).

## Discussion

This meta-analysis aimed to address the ongoing debate regarding the suitability of liver resection and transplantation for patients with BCLC stage B HCC. Our findings reveal that liver resection can be performed safely in selected patients, with a low mortality rate of less than 4% and a promising 5-year survival rate of 46%. However, the recurrence rate remains high. Future studies, particularly prospective designs, are warranted to refine patient selection criteria and optimize long-term outcomes after liver resection in this patient population.

Although liver resection is generally recommended for very early and early-stage HCC, its use for intermediate or advanced stages like BCLC B is controversial. This is mainly due to the concern of postoperative liver failure in patients with underlying liver cirrhosis. However, BCLC B patients often exhibit varying degrees of liver function impairment, making individual case evaluation crucial for determining eligibility for liver resection. This is supported by our findings, where 94% of patients who underwent resection had a Child-Pugh A score, and the reported median MELD score was below 10.

It is well established that patients with compensated liver cirrhosis tolerate liver resection well [14]. This finding aligns with the observed low mortality rate of 4% and major complication rate of 12% in our study. While Child-Pugh and MELD scores are valuable tools, they are not necessarily the best predictors of mortality after liver resection in patients with liver cirrhosis. Additional assessments like the albumin-bilirubin gradient, liver volume data, or dynamic liver function tests can provide more precise information on preoperative functional liver capacity. Unfortunately, such data was not available for this specific patient subset. However, considering the low complication rate, it is reasonable to assume that these patients exhibited preserved liver function. It is also noteworthy that a significant portion of the resections were categorized as minor (less than 3 segments), suggesting a preference for smaller and non-anatomical procedures.

Our analysis indicates that the included studies considered tumor extent for the selection of candidates for liver resection. BCLC B patients undergoing liver resection were outside the Milan criteria (one lesion < 5 cm or maximum of three lesion < 3 cm each). This finding is well expected considering that a single tumor of any size is classified as a BCLC A rather than BCLC B as recommended by the 2011 update. Patients were respectively classified as BCLC B based on multinodular disease with in the included studies with mean tumor number less than 3, while patients with tumor number more than 3 were not resected. Careful patient selection, typically encompassing individuals with preserved liver function, was observed in the analysis. Liver resection demonstrated favorable safety outcomes. This finding, along with a promising 5-year survival rate of 50%, reflects a favorable balance between perioperative risks and survival benefit. Notably, these outcomes surpass those of any reported palliative treatment options [7, 8]. It is worth noting that the 5-year survival rate observed here is comparable to that reported for BCLC stage 0-A resections [15]. 

Survival in HCC depends not only on tumor size and/or number, but also on tumor biology. The latter is not an integral part of the BCLC tumor staging system. The definition of the intermediate BCLC stage B is solely based on the number tumors, a fact which was challenged by several authors. Many previous reports have demonstrated that patients with favorable tumor biology can have a significant survival benefit despite the fact that an HCC exceeds the current morphologic limits for liver transplantation (e.g. Milan criteria) [3]. For example, it was reported to use AFP levels or a morphologic evaluation to assess tumor biology [16]. Results of positron emission tomography, response to downstaging with locoregional treatment are also useful to determine and select patients with favorable tumor biology. Those promising data in the literature were the ultimate reason to integrate liver transplantation in the treatment algorithm of BCLC stage B patients with favorable tumor biology. Currently, the updated version states TACE as the only treatment or bridging option, while liver resection could be a justified treatment option for patients with BCLC stage B HCC. Worth to note that the meta-analysis included one randomized controlled trial who observed an improved survival after liver resection compared to TACE in a combined analysis of BCLC B and BCLC C patients [2]. A subgroup analysis including only BCLC B patients confirmed the superiority of resection compared to TACE [2]. Nevertheless, liver resection cannot be recommended for every BCLC B patient and the choice between TACE and resection might be different across countries and health care systems. Countries with a transplant waiting time of only a few months may continue to choose TACE for bridging to transplantation or evaluation of the tumor biology. However, liver resection might be a reasonable strategy if liver transplantation is not available or the time on the waiting list is considerably long. Tumor recurrence in resected patients is however common. Although data on treatment of recurrence was not available for this study, the long-term results are indicative for further treatment of recurrence.

While recurrence rates are generally lower after liver transplantation due to the simultaneous treatment of the underlying liver disease, most centers do not classify patients undergoing extended criteria liver transplantation according to the BCLC system, even though they often apply extended criteria. This suggests that tumor biology may be considered the most important factor for extended criteria liver transplantation selection, while liver function and patient performance status may be potentially less emphasized, potentially due to being addressed by the transplant itself. This may explain uncommon use of the BCLC staging system in patients undergoing extended criteria liver transplantation for HCC.

This review has several limitations. First, all included studies were retrospective and therefore include a selection bias. Although mean number of tumors was 3, there were patients with multinodular disease > 4 which possibly have influenced the outcome. Since patients were selected carefully respecting tumor biology, liver function and performance status, findings cannot be generalized to all patients with BCLC stage B. In addition, overlapping treatment modalities cannot be excluded, since patients had other treatment modalities prior to or after to resection, e.g. TACE, for which the data was not available. The review was designed also to explore liver transplantation in BCLC stage B. However, only few studies were identified and the meta-analysis is based mostly on the available patient level data. Consequently, all results regarding the effectiveness of liver transplantation in this specific stage should be interpreted with caution. This meta-analysis aimed to evaluate the effectiveness of liver resection and transplantation for BCLC stage B. A direct comparison of the two procedures was not conducted on purpose. In our opinion, there is no evidence to support using case-control studies for comparing resection versus transplantation in this patient population. Indeed, none of the studies included in this meta-analysis reported data on both liver resection and transplantation for BCLC stage B. This finding supports our decision to focus solely on single-arm studies.

In conclusion, this meta-analysis revealed that liver resection can be performed safely in carefully selected BCLC stage B HCC patients, with a promising 5-year survival rate of 50% and a 5-year recurrence-free survival rate of 21%. Notably, the observed mortality rate was below 4%. These findings support the potential role of liver resection as a valuable treatment option and alternative for BCLC B patients, particularly those with preserved liver function. Additionally, liver resection might be a bridging strategy to transplantation in regions with extended waiting times. However, no conclusion can be done on liver transplantation in this stage due to lack data for meta-analysis.

## Electronic supplementary material

Below is the link to the electronic supplementary material.

Supplementary Material 1

Supplementary Material 2

Supplementary Material 3

## Data Availability

No datasets were generated or analysed during the current study.
