# Correction to: The role of resection in hepatocellular carcinoma BCLC stage B: A multi-institutional patient-level meta-analysis and systematic review

**DOI:** 10.1007/s00423-024-03518-2

**Published:** 2024-10-25

**Authors:** Victor Lopez-Lopez, Fabian Kalt, Jian-Hong Zhong, Cristiano Guidetti, Paolo Magistri, Fabrizio Di Benedetto, Arndt Weinmann, Jens Mittler, Hauke Lang, Rohini Sharma, Mathew Vithayathil, Samir Tariq, Patricia Sánchez-Velázquez, Gianluca Rompianesi, Roberto Ivan Troisi, Concepción Gómez-Gavara, Mar Dalmau, Francisco Jose Sanchez-Romero, Camilo Llamoza, Christoph Tschuor, Uluk Deniz, Georg Lurje, Peri Husen, Sandro Hügli, Jan Philipp Jonas, Fabian Rössler, Philipp Kron, Michaela Ramser, Pablo Ramirez, Kuno Lehmann, Ricardo Robles-Campos, Dilmurodjon Eshmuminov

**Affiliations:** 1 Department of General, Visceral and Transplantation Surgery, Clinic and University Hospital Virgen de La Arrixaca, IMIB-ARRIXACA, El Palmar, Murcia, Spain; 2 Department of Surgery and Transplantation, University Hospital Zurich and University of Zurich, Raemistrasse 100, Zurich, CH-8091 Switzerland; 3 Department of Hepatobiliary Surgery, Guangxi Medical University Cancer Hospital, Nanning, 530021 China; 4 Hepato-Pancreato-Biliary Surgery and Liver Transplantation Unit, University of Modena and Reggio Emilia, Modena, Italy; 5 Department of Internal Medicine I, University Medical Center of the Johannes Gutenberg University Mainz, Mainz, Germany; 6 Department of General, Visceral and Transplant Surgery, University Medical Center, Johannes Gutenberg-University Mainz, 55131 Mainz, Germany; 7 Department of Surgery and Cancer, Imperial College London, London, UK; 8 Division of Hepatobliary and pancreatic Surgery, Hospital del Mar, Universitat Pompeu Fabra, IMIM, Barcelona, Spain; 9 Department of Clinical Medicine and Surgery, Federico II University, Naples, Italy; 10 Department HPB and Transplantation Surgery, Hospital Vall d’Hebron, Universitat Autònoma de Barcelona, Barcelona, Spain; 11 Department of Surgery and Transplantation, Rigshospitalet Copenhagen University Hospital, Blegdamsvej 9 Copenhagen Ø, Copenhagen, 2100 Denmark; 12 Department of Surgery, Charité– Universitätsmedizin Berlin, corporate member of Freie Universität Berlin, Humboldt-Universität zu Berlin, Berlin Institute of Health, Berlin, Germany; 13 Department of General, Visceral and Transplantation Surgery, Heidelberg University Hospital, Heidelberg, Germany

**Correction to: Langenbeck’s Archives of Surgery (2024) 409:277**.

10.1007/s00423-024-03466-x.

This article unfortunately contained a mistake. The author name ‘Mathew Vithayathil’ was incorrectly written as ‘Vithayathil Mathew K.’.

The original article has been corrected.

