# Supplementary material for: The role of resection in hepatocellular carcinoma BCLC stage B: A multi-institutional patient-level meta-analysis and systematic review
Source: Langenbecks Arch Surg. 2024 Sep 13;409(1):277. doi: 10.1007/s00423-024-03466-x (PMC11399194; doi:10.1007/s00423-024-03466-x)
Supplement: Supplementary file 1 — Supplementary Material 1 [file 423_2024_3466_MOESM1_ESM.pdf]

Cookies
Our site uses cookies to improve your experience. You can find out more about our use of cookies in About Cookies, including instructions on how to turn off cookies if you wish to do so. By continuing to browse this site you agree to us using cookies as described in About Cookies.

I accept

Cochrane Library

Explore new Cochrane Library features **here**.

We noticed your browser language is German.
You can select your preferred language at the top of any page, and you will see translated Cochrane Review sections in this language. Change to **German.**

Advanced Search

Search manager

Save this search | View saved searches | Search help

View fewer lines | Print

#1	((hepatocellular OR liver OR "liver cell" OR "hepatic cell") NEAR/3 carcinoma*):ti,ab,kw OR (hepatocarcinoma OR hcc OR (malignant NEAR/3 hepatoma)):ti,ab,kw	5453
#2	(pugh OR BCLC OR "Barcelona Clinic Liver Cancer"):ti,ab,kw OR (child NEAR/3 B):ti,ab,kw	3364
#3	#1 AND #2	1245
#4	(hepatectom* OR segmentectom* OR sectionectom* OR hemi-hepat*):ti,ab,kw OR ((liver OR hepat*) NEAR/9 (resect*)):ti,ab,kw OR ((liver OR hepat* OR hcc) NEAR/6 (surg*)):ti,ab,kw	5430
#5	((liver or hepat* OR hcc) NEAR/6 (transplant* OR graft*)):ti,ab,kw	4857
#6	#4 OR #5	9423
#7	#3 AND #6	397
#8	Type a search term or use the S or MeSH buttons to compose	N/A

Clear all

Highlight orphan lines

Save this search | View saved searches | Search help

View fewer lines | Print

Filter your results

Cochrane Reviews 3 | Cochrane Protocols 0 | Trials 394 | Editorials 0 | Special collections 0 | Clinical Answers 0 | Other Reviews

For COVID-19 related studies, please also see the **Cochrane COVID-19 Study Register**

394 Trials matching **"#7 - #3 AND #6"**

Cochrane Central Register of Controlled Trials
Issue 7 of 12, July 2020

Deselect all (394) Export selected citation(s)

Order by Relevancy Results per page 25

1

Cytoreductive Surgery and Transarterial Chemoembolization (TACE) Versus TACE for Hepatocellular Carcinoma
NCT00820157
https://clinicaltrials.gov/show/NCT00820157, **2009** | added to CENTRAL: 31 May 2018 | 2018 Issue 5
CT.gov

2 ☑

Study of sorafenib and radiation therapy for advanced hepatocellular carcinoma with portal vein thrombosis
JPRN-UMIN000008184
http://www.who.int/trialsearch/Trial2.aspx?TrialID=JPRN-UMIN000008184, **2012** | added to CENTRAL: 31 March 2019 | 2019 Issue 3
ICTRP

3 ☑

Multimodal treatment modalities are associated with improved longterm outcome in patients with recurrent hepatocellular carcinoma
J Bednarsch, M Forster, W Schoning, T Ulmer, I Amygdalos, Z Czigany, DM Santana, F Meister, J Bocker, A Kroh, D Kroy, U Neumann, G Lurje
Zeitschrift fur gastroenterologie. Conference: 34. Jahrestagung der deutschen arbeitsgemeinschaft zum studium der leber deutsche arbeitsgemeinschaft zum studium der leber. Germany, **2018**, 56(1) (no pagination) | added to CENTRAL: 30 April 2018 | 2018 Issue 4
Embase

4 ☑

A prospective randomized controlled trial of selective DEB-epiDOX vs. selective conventional TACE for hepatocellular carcinoma focusing on local complete response rate
JPRN-UMIN000021250
http://www.who.int/trialsearch/Trial2.aspx?TrialID=JPRN-UMIN000021250, **2016** | added to CENTRAL: 31 March 2019 | 2019 Issue 3
ICTRP

5 ☑

Randomized comparative study of HCC with PVTT treated by interventional therapy and proton heavy ion radiotherapy and sorafenib combined with or without endovascular implantation of Iodine?125(125 I) seeds
ChiCTR-INR-17012083
http://www.who.int/trialsearch/Trial2.aspx?TrialID=ChiCTR-INR-17012083, **2017** | added to CENTRAL: 31 March 2019 | 2019 Issue 3
ICTRP

6 ☑

A Clinical Randomized Control Trial of Combination TACE With and Without Low-Molecular-Weight Heparin in Hepatocellular Carcinoma
ChiCTR-TRC-08000267
http://www.who.int/trialsearch/Trial2.aspx?TrialID=ChiCTR-TRC-08000267, **2008** | added to CENTRAL: 31 March 2019 | 2019 Issue 3
ICTRP

7 ☑

PhaseI/ II study of disulfiram in patients with advanced hepatocellular carcinoma
JPRN-UMIN000008529
http://www.who.int/trialsearch/Trial2.aspx?TrialID=JPRN-UMIN000008529, **2012** | added to CENTRAL: 31 March 2019 | 2019 Issue 3
ICTRP

8 ☑

Phase II study of sorafenib in patients with advanced hepatocellular carcinoma
JPRN-UMIN000002972
http://www.who.int/trialsearch/Trial2.aspx?TrialID=JPRN-UMIN000002972, **2010** | added to CENTRAL: 31 March 2019 | 2019 Issue 3
ICTRP

9 ☑

Randomized phase II study of sorafenib and hepatic arterial infusion chemotherapy with cisplatin versus sorafenib for advanced hepatocellular carcinoma
JPRN-UMIN000005703
http://www.who.int/trialsearch/Trial2.aspx?TrialID=JPRN-UMIN000005703, **2011** | added to CENTRAL: 31 March 2019 | 2019 Issue 3
ICTRP

10 ☑

Comparison of laproscopic and conventional hepatectomy for treatment of hepatocellular carcinoma: a randomized controlled trials
ChiCTR-INR-16009383
http://www.who.int/trialsearch/Trial2.aspx?TrialID=ChiCTR-INR-16009383, **2016** | added to CENTRAL: 31 March 2019 | 2019 Issue 3

ICTRP

11 ☑

Efficacy and safety of selective internal radiotherapy with yttrium-90 resin microspheres compared with sorafenib in locally advanced and inoperable hepatocellular carcinoma (SARAH): an open-label randomised controlled phase 3 trial

V Vilgrain, H Pereira, E Assenat, B Guiu, AD Ilonca, G-P Pageaux, A Sibert, M Bouattour, R Lebtahi, W Allaham, H Barraud, V Laurent, E Mathias, J-P Bronowicki, J-P Tasu, R Perdrisot, C Silvain, R Gerolami, O Mundler, J-F Seitz, V Vidal, C Aube, F Oberti, O Couturier, I Brenot-Rossi, J-L Raoul, A Sarran, C Costentin, E Itti, A Luciani, R Adam, M Lewin, D Samuel, M Ronot, A Dinut, L Castera, G Chatellier

Lancet oncology, **2017**, (no pagination) | added to CENTRAL: 30 November 2017 | 2017 Issue 11

Embase

12 ☑

Efficacy and safety of selective internal radiotherapy with yttrium-90 resin microspheres compared with sorafenib in locally advanced and inoperable hepatocellular carcinoma (SARAH): an open-label randomised controlled phase 3 trial

V Vilgrain, H Pereira, E Assenat, B Guiu, AD Ilonca, GP Pageaux, A Sibert, M Bouattour, R Lebtahi, W Allaham, H Barraud, V Laurent, E Mathias, JP Bronowicki, JP Tasu, R Perdrisot, C Silvain, R Gerolami, O Mundler, JF Seitz, V Vidal, C Aubé, F Oberti, O Couturier, I Brenot-Rossi, JL Raoul, A Sarran, C Costentin, E Itti, A Luciani, R Adam, M Lewin, D Samuel, M Ronot, A Dinut, L Castera, G Chatellier

The lancet. Oncology, **2017**, 18(12), 1624-1636 | added to CENTRAL: 31 January 2018 | 2018 Issue 1

PubMed

13 ☑

Fast-track surgery in hepatocellular carcinoma following open liver resection: a prospective randomized controlled trial

ChiCTR-TRC-14004946

http://www.who.int/trialsearch/Trial2.aspx?TrialID=ChiCTR-TRC-14004946, **2014** | added to CENTRAL: 31 March 2019 | 2019 Issue 3

ICTRP

14 ☑

Prospective study of efficacy and safety of transcatheter arterial chemoembolization (TACE) with miriplatin for hepatocellular carcinoma

JPRN-UMIN000005995

http://www.who.int/trialsearch/Trial2.aspx?TrialID=JPRN-UMIN000005995, **2011** | added to CENTRAL: 31 March 2019 | 2019 Issue 3

ICTRP

15 ☑

Heating effect of miriplatin on transcatheter arterial chemoembolization therapy for hepatocellular carcinoma: a randomized controlled trial

JPRN-UMIN000010605

http://www.who.int/trialsearch/Trial2.aspx?TrialID=JPRN-UMIN000010605, **2013** | added to CENTRAL: 31 March 2019 | 2019 Issue 3

ICTRP

16 ☑

RCT of Different Effects of Nucleot(s)Ide Analogues on the Prognosis of HBV-HCC Patients After Curative Resection

NCT04032860

https://clinicaltrials.gov/show/NCT04032860, **2019** | added to CENTRAL: 31 August 2019 | 2019 Issue 08

CT.gov

17 ☑

Natural history of untreated nonsurgical hepatocellular carcinoma: rationale for the design and evaluation of therapeutic trials

JM Llovet, J Bustamante, A Castells, R Vilana, C Ayuso Mdel, M Sala, C Brú, J Rodés, J Bruix

Hepatology (baltimore, md.), **1999**, 29(1), 62-67 | added to CENTRAL: 31 January 1998 | 1998 Issue 1

PubMed

18 ☑

Prospective randomized trial of preoperative transcatheter arterial chemoembolization for resectable large hepatocellular carcinoma

JPRN-UMIN000005241

http://www.who.int/trialsearch/Trial2.aspx?TrialID=JPRN-UMIN000005241, **2011** | added to CENTRAL: 31 March 2019 | 2019 Issue 3

ICTRP

19 ☑

Randomized controlled trial for proton beam radiotherapy versus transarterial chemoembolization for the treatment of hepatocellular carcinoma; Preliminary results

Z Kayali, J Slater, D Bush

Journal of hepatology, **2013**, 58, S111- | added to CENTRAL: 31 October 2014 | 2014 Issue 10

Embase

20 ☑

Feasibility and efficacy of high-dose three-dimensional-conformal radiotherapy in cirrhotic patients with small-size hepatocellular carcinoma non-eligible for curative therapies--mature results of the French Phase II RTF-1 trial

F Mornex, N Girard, C Beziat, A Kubas, M Khodri, C Trepo, P Merle

International journal of radiation oncology, biology, physics, **2006**, 66(4), 1152-1158 | added to CENTRAL: 31 October 2008 | 2008 Issue 4

PubMed

21 ☑

A prospective clinical trial on sorafenib treatment of hepatocellular carcinoma before liver transplantation

MS Eilard, M Andersson, P Naredi, C Geronymakis, P Lindner, C Cahlin, W Bennet, M Rizell

BMC cancer, **2019**, 19(1) | added to CENTRAL: 31 August 2019 | 2019 Issue 08

PubMed Embase

22 ☑

HCC: ablation techniques

T Bilhim

Cardiovascular and interventional radiology, **2016**, 39(3), S64-S65 | added to CENTRAL: 28 February 2017 | 2017 Issue 2

Embase

23 ☑

A RCT on the patients with hepatocellular carcinoma receiving chemotherapy of arsenic trioxide for preventing tumor recurrence after hepatectomy

ChiCTR-TRC-13003244

http://www.who.int/trialsearch/Trial2.aspx?TrialID=ChiCTR-TRC-13003244, **2013** | added to CENTRAL: 31 March 2019 | 2019 Issue 3

ICTRP

24 ☑

Surgery With or Without Hepatic Arterial Chemotherapy in Treating Patients With Liver Cancer

NCT00238160

https://clinicaltrials.gov/show/NCT00238160, **2005** | added to CENTRAL: 31 May 2018 | 2018 Issue 5

CT.gov

25 ☑

A randomized controlled trial to compare the effectiveness between surgery and radiofrequency ablation for hepatocellular carcinoma: SURF trial

K Hasegawa, N Kokudo, Y Matsuyama

Journal of clinical oncology, **2017**, 35(4) | added to CENTRAL: 30 November 2017 | 2017 Issue 11

Embase

1 2 3 4 5 6 7 8 9 10 11 Next

Embase®

Embase Session Results (2 Jul 2020)

No.	Query	Results
#10	#3 AND #6 NOT [conference abstract]/lim AND [english]/lim NOT ([editorial]/lim OR [letter]/lim OR [note]/lim)	2819
#9	#3 AND #6 NOT [conference abstract]/lim AND [english]/lim	2942
#8	#3 AND #6 NOT [conference abstract]/lim	3147
#7	#3 AND #6	5126
#6	#4 OR #5	234210
#5	'liver transplantation'/exp OR (((liver OR hepat* OR hcc) NEAR/6 (transplant* OR graft*)):ti,ab,kw)	142089
#4	'liver cell carcinoma'/exp/dm_su OR 'liver resection'/exp OR hepatectom*:ti,ab,kw OR segmentectom*:ti,ab,kw OR sectionectom*:ti,ab,kw OR 'hemi hepat*':ti,ab,kw OR (((liver OR hepat*) NEAR/9 resect*):ti,ab,kw) OR (((liver OR hepat* OR hcc) NEAR/6 surg*):ti,ab,kw)	114123
#3	#1 AND #2	12898
#2	'child pugh score'/exp OR pugh:ti,ab,kw OR bclc:ti,ab,kw OR 'barcelona clinic liver cancer':ti,ab,kw OR ((child NEAR/3 b):ti,ab,kw)	25725
#1	'liver cell carcinoma'/exp OR (((hepatocellular OR liver OR 'liver cell' OR 'hepatic cell') NEAR/3 carcinoma*):ti,ab,kw) OR hepatocarcinoma:ti,ab,kw OR hcc:ti,ab,kw OR ((malignant NEAR/3 hepatoma):ti,ab,kw)	193613

ELSEVIER

© 2020 Elsevier Life Sciences IP Limited except certain content provided by third parties.
Embase is a trade mark of Elsevier Life Sciences IP Limited. RELX Group and the RE symbol are trade marks of RELX Group plc, used under license.

Ovid®

Wolters Kluwer

Support & Training Feedback Close

Database(s): **Ovid MEDLINE(R) and Epub Ahead of Print, In-Process & Other Non-Indexed Citations and Daily** 1946 to June 30, 2020

Search Strategy:

#	Searches	Results
1	("31499131" or "29510616" or "26734621" or "29372372" or "29503583" or "29988922").ui.	6
2	exp Carcinoma, Hepatocellular/ or ((hepatocellular or liver or "liver cell" or "hepatic cell") adj3 carcinoma*).mp. or (hepatocarcinoma or hcc or (malignant adj3 hepatoma)).mp.	125384
3	(pugh or BCLC or "Barcelona Clinic Liver Cancer" or (child adj3 B)).mp.	10733
4	2 and 3	4856
5	exp Carcinoma, Hepatocellular/su or exp Hepatectomy/ or (hepatectom* or segmentectom* or sectionectom* or hemi-hepat*).mp. or ((liver or hepat*) adj9 resect*).mp. or ((liver or hepat* or hcc) adj6 surg*).mp.	76103
6	exp Liver Transplantation/ or exp Carcinoma, Hepatocellular/tr or ((liver or hepat* or hcc) adj6 (transplant* or graft*)).mp.	86427
7	5 or 6	149464
8	4 and 7	1952
9	limit 8 to (abstracts and english language)	1788
10	1 and 9	6

1. Development of a nomogram to predict outcome after **liver** **resection** for **hepatocellular** **carcinoma** in Child-Pugh **B** cirrhosis.

   Berardi G; Morise Z; Sposito C; Igarashi K; Panetta V; Simonelli I; Kim S; Goh BKP; Kubo S; Tanaka S; Takeda Y; Ettorre GM; Wilson GC; Cimino M; Chan CY; Torzilli G; Cheung TT; Kaneko H; Mazzaferro V; Geller DA; Han HS; Kanazawa A; Wakabayashi G; Troisi RI.

   Journal of Hepatology. 72(1):75-84, 2020 Jan.

   [Journal Article]

   UI: 31499131

   Authors Full Name

   Berardi, Giammauro; Morise, Zenichi; Sposito, Carlo; Igarashi, Kazuharu; Panetta, Valentina; Simonelli, Ilaria; Kim, Sungho; Goh, Brian K P; Kubo, Shoji; Tanaka, Shogo; Takeda, Yutaka; Ettorre, Giuseppe Maria; Wilson, Gregory C; Cimino, Matteo; Chan, Chung-Yip; Torzilli, Guido; Cheung, Tan To; Kaneko, Hironori; Mazzaferro, Vincenzo; Geller, David A; Han, Ho-Seong; Kanazawa, Akishige; Wakabayashi, Go; Troisi, Roberto Ivan.

   Cite

Brought to you by UZH Hauptbibliothek / Zentralbibliothek Zürich

Advanced search

Compare sources

Documents Authors Affiliations Advanced

Search tips

Enter query string

TITLE-ABS-KEY((liver or hepat* OR hcc) W/6 (transplant* OR graft*))

Outline query Add Author name / Affiliation Clear form

ALL("Cognitive architectures") AND AUTHOR-NAME(smith)
TITLE-ABS-KEY(*somatic complaint wom?n) AND PUBYEAR AFT 1993
SRCTITLE(*field ornith*) AND VOLUME(75) AND ISSUE(1) AND PAGES(53-66)

Operators

Field codes

Search history

7 (TITLE-ABS-KEY ((hepatocellular OR liver OR "liver cell" OR "hepatic cell") W/3 carcinoma*) OR TITLE-ABS-KEY (hepatocarcinoma OR hcc OR (malignant W/3 hepatoma))) AND (TITLE-ABS-KEY (pugh OR bclc OR "Barcelona Clinic Liver Cancer") OR TITLE-ABS-KEY (child PRE/3 b)) AND ((TITLE-ABS-KEY (hepatectom* OR segmentectom* OR sectionectom* OR hemi-hepat*) OR TITLE-ABS-KEY ((liver OR hepat*) W/9 (resect*)) OR TITLE-ABS-KEY ((liver OR hepat* OR hcc) W/6 (surg*))) OR (TITLE-ABS-KEY ((liver OR hepat* OR hcc) W/6 (transplant* OR graft*)))) AND (EXCLUDE (DOCTYPE , "le") OR EXCLUDE (DOCTYPE , "ed") OR EXCLUDE (DOCTYPE , "no") OR EXCLUDE (DOCTYPE , "sh")) AND (LIMIT-TO (LANGUAGE , "English")) — 2,509 document results

6 (TITLE-ABS-KEY ((hepatocellular OR liver OR "liver cell" OR "hepatic cell") W/3 carcinoma*) OR TITLE-ABS-KEY (hepatocarcinoma OR hcc OR (malignant W/3 hepatoma))) AND (TITLE-ABS-KEY (pugh OR bclc OR "Barcelona Clinic Liver Cancer") OR TITLE-ABS-KEY (child PRE/3 b)) AND ((TITLE-ABS-KEY (hepatectom* OR segmentectom* OR sectionectom* OR hemi-hepat*) OR TITLE-ABS-KEY ((liver OR hepat*) W/9 (resect*)) OR TITLE-ABS-KEY ((liver OR hepat* OR hcc) W/6 (surg*))) OR (TITLE-ABS-KEY ((liver OR hepat* OR hcc) W/6 (transplant* OR graft*)))) AND (EXCLUDE (DOCTYPE , "le") OR EXCLUDE (DOCTYPE , "ed") OR EXCLUDE (DOCTYPE , "no") OR EXCLUDE (DOCTYPE , "sh")) — 2,696 document results

5 (TITLE-ABS-KEY ((hepatocellular OR liver OR "liver cell" OR "hepatic cell") W/3 carcinoma*) OR TITLE-ABS-KEY (hepatocarcinoma OR hcc OR (malignant W/3 hepatoma))) AND (TITLE-ABS-KEY (pugh OR bclc OR "Barcelona Clinic Liver Cancer") OR TITLE-ABS-KEY (child PRE/3 b)) AND ((TITLE-ABS-KEY (hepatectom* OR segmentectom* OR sectionectom* OR hemi-hepat*) OR TITLE-ABS-KEY ((liver OR hepat*) W/9 (resect*)) OR TITLE-ABS-KEY ((liver OR — 2,836 document results

hepat* OR hcc) W/6 (surg*))) OR (TITLE-ABS-KEY ((liver OR hepat* OR hcc) W/6 (transplant* OR graft*))))

4	TITLE-ABS-KEY ((liver OR hepat* OR hcc) W/6 (transplant* OR graft*))	106,594 document results	
3	TITLE-ABS-KEY (hepatectom* OR segmentectom* OR sectionectom* OR hemi-hepat*) OR TITLE-ABS-KEY ((liver OR hepat*) W/9 (resect*)) OR TITLE-ABS-KEY ((liver OR hepat* OR hcc) W/6 (surg*))	92,514 document results	
2	TITLE-ABS-KEY (pugh OR bclc OR "Barcelona Clinic Liver Cancer") OR TITLE-ABS-KEY (child PRE/3 b)	20,530 document results	
1	TITLE-ABS-KEY ((hepatocellular OR liver OR "liver cell" OR "hepatic cell") W/3 carcinoma*) OR TITLE-ABS-KEY (hepatocarcinoma OR hcc OR (malignant W/3 hepatoma))	167,205 document results	

Showing all recent searches | View 5 most recent only

Top of page

Help improve Scopus

About Scopus

What is Scopus
Content coverage
Scopus blog
Scopus API
Privacy matters

Language

日本語に切り替える
切換到简体中文
切換到繁體中文
Русский язык

Customer Service

Help
Contact us

Terms and conditions Privacy policy

Copyright © Elsevier B.V. All rights reserved. Scopus® is a registered trademark of Elsevier B.V.

We use cookies to help provide and enhance our service and tailor content. By continuing, you agree to the use of cookies.
