# Supplementary material for: The role of resection in hepatocellular carcinoma BCLC stage B: A multi-institutional patient-level meta-analysis and systematic review
Source: Langenbecks Arch Surg. 2024 Sep 13;409(1):277. doi: 10.1007/s00423-024-03466-x (PMC11399194; doi:10.1007/s00423-024-03466-x)
Supplement: Supplementary file 2 — Supplementary Material 2 [file 423_2024_3466_MOESM2_ESM.docx]

| **Concluded Evidences** | **Meta-analysis** | **Level of Evidence** |
| --- | --- | --- |
| **A Liver resection** |  |  |
| - Median overall survival | 50 months (95% CI 38-62 months) | Low |
| - Recurrence-free survival | 15 months (95% CI 12-18 months) | Low |
| - Microvascular invasion | 0.42 (95% CI, 0.34-0.51; I^2^=66%, p<0.01) | Very Low |
| - Macrovascular invasion | 0.08 (95% CI, 0.03-0.21; I^2^=63%, p=0.01) | Very Low |
| - R0 resection | 0.92 (95%-CI, 0.82-0.97; I^2^=83%, p<0.01) | Very Low |
| - Total complications | 0.46 (95% CI, 0.34-0.58; I^2^=92%, p<0.01) | Very Low |
| - Major complications | 0.11 (95% CI, 0.0-0.17; I^2^=84%, p<0.01) | Very Low |
| - 90-day mortality rate | 0.03 (95% CI, 0.03-0.08; I^2^=58%, p<0.01) | Very Low |
| - Minor resection rate | 0.60 (95% CI, 0.5-0.67; I^2^=78%, p<0.01) | Very Low |
| - Child-Pugh A | 0.93 (95% CI, 0.90-0.96; I^2^=90%, p<0.01) | Very Low |
| - The mean tumor number | 2.88 (95% CI, 1.69-4.07; I^2^=0, p=0.85) | Low |
| - Largest tumor size | 6.85 cm (95% CI, 2.96-10.74; I^2^=0, p=0.99) | Low |
|  |  |  |
| **B Liver Transplantation** |  |  |
| - Median overall survival | Not reached | Very Low |
| - Recurrence-free survival | Not reached | Very Low |
| - Microvascular invasion | 0.11 (95% CI, 0.02-0.44; I^2^=69%, p=0.04) | Very Low |
| - Macrovascular invasion | 0.05 (95% CI, 0.01-0.2; I^2^=60%, p=0.08) | Very Low |
| - Total complications | 0.57 (95% CI, 0.45-0.68; I^2^=0, p=0.41) | Very Low |
| - Major complications | 0.13 (95% CI, 0.04-0.36; I^2^=63%, p=0.07) | Very Low |
| - 90-day mortality rate | 0.06 (95% CI, 0.03-0.12; I^2^=0, p=0.84) | Very Low |
| - Child-Pugh A | 0.60 (95% CI, 0.43-0.74; I^2^=62%, p=0.05) | Very Low |
| - The mean tumor number | 3.19 (95% CI, 1.41-4.96; I^2^=0, p=0.84) | Very Low |
| - Largest tumor size | 4.72cm (95% CI, 1.62-7.82; I^2^=0, p=0.94) | Very Low |

**Supplementary Table 2 GRADE evidences.**
