# Supplementary material for: The role of resection in hepatocellular carcinoma BCLC stage B: A multi-institutional patient-level meta-analysis and systematic review
Source: Langenbecks Arch Surg. 2024 Sep 13;409(1):277. doi: 10.1007/s00423-024-03466-x (PMC11399194; doi:10.1007/s00423-024-03466-x)
Supplement: Supplementary file 3 — Supplementary Material 3 [file 423_2024_3466_MOESM3_ESM.docx]

| **Author / year** | **Selection** | **Comparability** | **Outcome** | **Total** |
| --- | --- | --- | --- | --- |
| Bell et al. 2016(17) | *** | * | *** | 7* |
| Di Sandro et al. 2019(18) | *** | * | *** | 7* |
| Fang et al. 2019(19) | ** | ** | * | 5* |
| Garancini et al. 2017(20) | ** | ** | * | 5* |
| Lei et al. 2014(21) | *** | ** | *** | 8* |
| Kamiyama et al. 2017(22) | ** | ** | *** | 7* |
| Kamo et al. 2018(23) | * | ** | *** | 6* |
| Kariyama et al. 2020(24) | *** | * | *** | 6* |
| Kim H et al. 2017(25) | *** | ** | *** | 8* |
| Kim J et al. 2016(26) | *** | * | *** | 8* |
| Lin C. T. et al. 2010(27) | *** | ** | ** | 7* |
| Lin C. W. et al. 2020(28) | *** | * | ** | 6* |
| Liu Y. et al. 2020(29) | * | * | *** | 5* |
| Matsukuma et al. 2018(30) | *** | * | *** | 7* |
| Peng et al. 2019(31) | *** | * | *** | 6* |
| Renner et al. 2015(32) | *** | * | *** | 6* |
| Torzilli et al. 2008(33) | *** | ** | *** | 8* |
| Tsilimigras et al. 2019(34) | *** | * | *** | 7* |
| Wada et al. 2016(35) | *** | * | ** | 6* |
| Wang et al. 2016(36) | *** | * | *** | 7* |
| Wei S. et al. 2011(37) | *** | * | *** | 7* |
| Wei W. et al. 2018(38) | **** | * | *** | 8* |
|  |  |  |  |  |
| Berardi et al. 2019(39) | *** | ** | *** | 8* |
| Di Benedetto et al. 2023 (40) | **** | ** | *** | 9* |
| Lim et al. 2018(41) | *** | * | *** | 7* |
| Lopez-Lopez et al. 2021(42) | *** | * | *** | 7* |
| Ramasvami et al. 2016(43) | ** | * | *** | 6* |
| Villamonte et al 2022(44) | *** | * | *** | 7* |
| Weinmann et al. / 2015(45) | **** | * | *** | 8* |
| Zhong et al. / 2014(9) | **** | * | *** | 8* |
| Charité, Berlin |  |  |  |  |

Supplementary Table 3. The publication bias according to Newcastle–Ottawa quality assessment scale.
